# An observational study of recess quality and physical activity in urban primary schools

**DOI:** 10.1186/s12889-020-08849-5

**Published:** 2020-05-27

**Authors:** William V. Massey, Megan B. Stellino, John Geldhof

**Affiliations:** 1 College of Public Health and Human Sciences, School of Biological and Population Health Sciences, Oregon State University, Corvallis, Oregon, USA; 2 School of Sport and Exercise Science, University of Northern Colorado, Greeley, CO USA; 3 College of Public Health and Human Sciences, School of Social and Behavioral Health Sciences, Oregon State University, Corvallis, Oregon, USA

**Keywords:** School health, Adult engagement, Play, Obesity

## Abstract

**Background:**

To date, there is scant literature that examines the recess context concurrent with, but separate from, levels of physical activity. The primary purpose of the current study was to examine how recess quality impacted physical activity levels, and how this was moderated by gender. A secondary purpose was to examine if differences in children’s engagement in activities occurred between recess sessions scored as low- or high- quality.

**Methods:**

This was an observational study of children at 13 urban elementary schools in the U.S. Across the 13 schools, data were collected at 55 recess sessions, with 3419 child-level observations (*n =* 1696 boys; *n =* 1723 girls). Physical activity data were collected using Fitbit accelerometers, recess quality data were collected using the Great Recess Framework – Observational Tool (GRF-OT), recess engagement data were collected using the Observation of Playground Play (OPP), and basic psychological need satisfaction (BPNS) data were collected using a modified version of the BPNS for recess physical activity survey. Primary analyses were conducted using Hierarchical Linear Modeling (HLM) with children nested within recess sessions.

**Results:**

Gender moderated the relationship between adult engagement and moderate-to-vigorous physical activity (MVPA) (b = .012; 95% CI .001, .024), student behavior and MVPA (b = −.014; 95% CI −.021, −.007), and student behaviors and light physical activity (b = .009, 95% CI .003, .015). Both boys and girls engaged in more play during recess sessions scored as high quality on the GRF-OT. Children reported higher levels of basic psychological need satisfaction at recesses sessions scored as high quality on the GRF-OT.

**Conclusions:**

Results of the current study showed that the quality of the recess environment, and the interactions of both adults and students in that environment, need to be taken into consideration in future school-based recess studies.

## Background

Low levels of physical activity (PA) remain a problem that contribute to the high obesity rates seen in children. Increasingly, schools have become a focus for physical activity interventions amongst researchers [1] particularly given the amount of time children spend in this environment. Unfortunately, children in the United States (U.S.) continue to lack meaningful opportunities for physical activity at school, despite research that shows time spent engaging in physical activity makes positive contributions to academics [2]. Aside from PE, school-based recess is also a prime opportunity for physical activity within the school day. Specifically, recess has been shown to account for 42% of children’s opportunities to be physically active in school [3], and up to 44% of step counts during the school day [4]. Despite this, data from the 2012–2013 academic year in the United States (U.S.) suggest that 60% of school districts have no formal policy regarding daily recess. Of school-districts that have a recess policy, only 22% require daily recess for elementary school students, with less than half of these requiring at least 20 min of recess per day [5]. While recent legislative efforts have been made to promote recess at the state level, only seven out of 50 U.S. states require daily recess for children during the school day [6].

Perhaps more concerning is the trends described above disproportionally affect children from disadvantaged backgrounds. Data show that children who go to large, urban schools; schools with a high minority population; and schools with low-income levels are the least likely to get access to recess, and often report the shortest amount of time dedicated to recess [7, 8]. It is plausible that environmental factors affect children’s access to physical activity opportunities in urban and low-income school systems. Notably, bullying and aggressive behavior have been reported on the playground at urban elementary schools [9, 10], which could result in fewer opportunities for students. Furthermore, access to space and equipment are thought to be central to recess facilitation [11], which could be lacking at low-income, and/or urban schools. For example, a systematic review conducted by Erwin et al. [4] suggests that adding more playground equipment and providing a structured recess yields the largest effect on physical activity during recess. Yet, budgetary restraints could limit the purchase of equipment in low-income school districts. Thus, there is a need to consider both access to recess, and the quality of the recess environment for promoting physical activity throughout the school day, particularly in low-income and urban environments.

Aside from addressing disparities to physical activity opportunities such as recess, a need also exists to examine barriers to, and facilitators for, physical activity when children do have access to discretionary time during the school day. One common area of focus has been on differences between activity levels for boys and girls, given that data have consistently shown that girls are less active during recess periods [12–14]. These data suggest that social determinants might play a role in children’s behavior on the playground. In examining barriers to physical activity, Pawlowski and colleagues [15, 16] reported several gendered issues that may limit physical activity during recess. For example, elementary school girls reported wanting to play sports (i.e., ball games), but those were usually dominated by the boys on the playground [15]. Moreover, activities at recess can be labeled along gender lines, with girls being expected to conform to more sedentary activities [16]. While girls seem more interested in crossing gender borders at recess [17], boys in the Pawlowski et al. study [16] dominated the larger playground spaces (i.e., football pitch) often excluding girls during the game. Thus, it appears that there is a need for both variety of games and play spaces, as well as inclusive behavior on the playground, in an effort to more effectively promote physical activity at recess. Indeed, researchers have reported girls engage in similar levels of moderate-to-vigorous physical activity (MVPA) as boys when playing team sports [13] and that providing an activity of the week intervention can yield gains in physical activity [18].

Another important consideration for examining behavior at recess is how perceptions of physical and emotional safety might impact physical activity behavior during recess. Previous research with child participants has suggested that conflict is a regular part of the playground experience [19, 20]. Similarly, behavioral observations have suggested that bullying regularly takes place during recess periods [10]. In terms of physical activity during recess, children have specifically discussed conflict as a barrier to play for boys and girls alike. Moreover, children have reported that a stronger presence by adults at recess might help to facilitate higher levels of play, as there would be a more neutral mediator to weigh in on games [16]. This notion was supported by a recent study that showed adult engagement and supervision as a significant predictor of play for boys and girls during recess [21]. Additional work has shown that the presence of recess interventions changes play patterns, specifically reducing non-engagement and increasing engagement in more traditional games such as four-square, hopscotch, and use of loose equipment [22].

In considering the potential barriers to physical activity at recess, multiple interacting factors are at play. Access to resources, school policies, the physical environment, various social determinants, and student behaviors all play various roles in facilitating, or impeding, physical activity at recess. Understanding optimal standards for facilitating physical activity during recess is currently needed, especially for those in urban and/or low-income schools who may have limited opportunities. To date, there is scant literature that examines the recess context concurrent with, but separate from, levels of physical activity. Recently, Massey and colleagues [23] developed an observational tool to measure environmental and social determinants of elementary school recess. Specifically, the Great Recess Framework – Observational Tool (GRF-OT) measures the safety and structure of the playground, adult engagement and supervision, as well as student behaviors. Given the overlap between the GRF-OT and research examining potential barriers and facilitators to recess physical activity, there is a need to examine how the above-mentioned contextual factors, as measured by the GRF-OT, impact physical activity levels. As such, the primary purpose of the current study was to examine how recess quality impacted physical activity levels, and how this was moderated by gender. As previous research has shown that physical activity at recess might not translate into prosocial engagement [10, 19], a secondary purpose was to examine if differences in children’s engagement in specific types of play (e.g., organized games, traditional playground activities, non-engagement in play) occurred between recess sessions observed as low- or high- quality.

## Methods

Institutional Review Board approval was obtained prior to the start of any study procedures (Concordia University Wisconsin ID: 932380–3; 926,512–1). Additionally, all protocols and procedures were approved by the research board at the school district level, as well as the principal at each school. In line with local policies, recruitment was done with the support of the district administrative team in an effort to produce a diverse sample representative of the school district. Following district approval, the PI contacted the principal of each school with an invitation to participate in the study. In an effort to ensure we could include a maximum number of children on the playground, a passive consent protocol was followed. Consent forms were sent home to each student and parents and students were given the opportunity to opt out of the current study. In accordance with school district policy on passive consent protocols, personally identifying information was not collected on participants.

### Participants

Participants included children at 13 urban elementary schools in the U.S. Across the 13 schools, accelerometer data were collected at 55 recess sessions, with 3419 child-level observations (*n =* 1696 boys; *n =* 1723 girls). Observations of engagement in recess activities were collected at 61 recess sessions with 4528 child-level observations (*n =* 2243 boys; *n =* 2285 girls). The number of children within each recess session ranged from 12 to 117 with an average of 62.16 (*SD =* 26.34) children per recess. All schools enrolled in this study served children in low-income areas, with publicly available data showing that 78.8% of the student population is economically disadvantaged (12-out-of-13 schools > 50% of economically disadvantaged students; range = 22.5–99.6%). Of the 13 schools, 10 were in the public-school system, with three of these 10 schools being language immersion schools. Twelve of the 13 schools were exclusively elementary schools (i.e., grades 1–5), and one school also served middle school students (i.e., grades 4–8). Enrollment at each school ranged from 253 students to 690 students, with an average of 436 students per school.

### Measures

#### Physical activity

The Fitbit Flex™ is a wrist worn triaxial accelerometer that uses proprietary algorithms to estimate steps counts and time spent in various activity levels. The Fitbit Flex provides the most simplistic user display of all Fitbit products, with only LED lights to represent progress towards daily goals (the default setting is 10,000 steps; 2000 steps per dot shown). This was thought to be advantageous, as participants would not be able to directly monitor their step counts of physical activity levels during recess. The Fitbit Flex can by synched wirelessly to a smart phone or tablet, and provides information on steps counts and time spend in various activity levels (i.e., sedentary, light, moderate, vigorous). For the purposes of the current study, the research team created anonymous accounts for each device that could only be accessed by the research team. Each account was assigned to either a male or female user, with national averages for height and weight being used for user demographic information. Fitbits were placed on students in their classroom, or in the lunch room, approximately 30 min prior to the start of recess. Data assessors recorded the exact start and stop times of recess so that data could be extracted to match the time stamp. Data were housed by a third-party vendor (Fitabase LLC, San Diego, California) and processed using 60 s epochs within the noted time stamp, the most sensitive setting available for this device. In child-based studies, both waist-worn [24] and wrist worn [25] Fitbit devices (Fitbit One and Fitbit Charge, respectively) have been shown to have consistent levels of step counts with Actigraph accelerometers, yet may over-estimate absolute number of steps, as well as time spent in MVPA. Additional research in young adult populations has shown moderate validity between the wrist-worn Fitbit Flex and the wrist-worn Actigraph GT3X+ in free-living conditions [26], yet the Fitbit flex showed higher levels of variability, and was more likely to under-estimated activity levels.

#### Recess quality

The Great Recess Framework – Observational Tool (GRF-OT) was used as a measure of recess quality in the current study. The GRF-OT represents four domains of recess that include safety and structure of the playground, adult supervision and engagement, student behaviors, and transitions to and from the recess space [23]. In the current study data was collected on three of the four sub-scales (transitions were excluded, as they account for the times immediately before and after recess and the focus of the current study was PA during recess). Items are scored on a 4-point scale by a live observer who was present at recess (4 = highest quality; 1 = lowest quality). The safety and structure sub-scale examines the physical environment and access to equipment; the adult engagement and supervision sub-scale examines the number of adults present, their proximity to students, and whether or not they engage with students on the playground; and the student behaviors sub-scales examines student engagement, initiation of play, conflict, and conflict resolution. Each item and its associated scoring procedure can be found in Massey et al. 2018 [20]. A complete scoring manual with detailed instructions is available at https://www.playworks.org/resources/great-recess-framework/. Data in the current study suggest acceptable levels of internal consistency for the safety and structure sub-scale (*α* = .806), adult engagement and supervision sub-scale (*α* = .736), and student behavior sub-scale (*α* = .788). Previous research has shown support for the inter-rater reliability and factorial validity of the GRF-OT [23].

#### Engagement in recess activities

The different types of activities children engaged in during recess were measured using the Observations of Playground Play (OPP) [22]. The OPP allows observes to code engagement in 32 common recess activities across eight different play domains. Observers are also able to write in observed behaviors within each of the eight domains to ensure all recess activities are captured. Previous research has been reported on the reliability of this assessment tool [22].

#### Basic psychological needs satisfaction (BPNS)

A sub-sample of fourth and fifth grade students (*n =* 820) completed a modified version of the basic psychological need satisfaction scale [27]. The original 21-item questionnaire designed to assess individual perceptions of autonomy (7 items), competence (6 items), and relatedness (8 items) need satisfaction at work was modified to specifically examine children’s need satisfaction during recess. Given the younger population, along with school district requirements around classroom disruptions for research purposes, the measure was reduced to four items. One of these items was added to the original scale and was related to safety at recess. All responses corresponded to a 5-point Likert scale (5 = high need satisfaction) on items such as “I feel forced to do things I don’t want to do at recess.” (autonomy), “When I’m playing at recess I often do not feel I’m good at physically active things” (competence), “I really like the kids I play with at recess” (relatedness), and “I am safe when I am playing at recess” (safety). The modified scale showed acceptable levels of measurement validity (χ^2^= 8.39, *p* = .015; CFI = .951; RMSEA = .064; SRMR = .025). Validity of the reduced measure of children’s recess physical activity basic psychological needs satisfaction was also considered sufficient for the scope of this study based on the following three criteria; (1) previous research results that demonstrated adequate psychometrics for the entire BPNS - modified for recess physical activity [27]; (2) the face validity evident by the explicit wording of the items employed; and (3) clear connection to theoretical contentions of Basic Needs Theory, a mini-theory of Self-Determination Theory (SDT: Deci & Ryan, 2017) upon which the original measure and selected specific items in the present reduced measure are based.

### Procedures

With the exception of one school that conducted concurrent indoor and outdoor recess periods, all recess data collection periods were conducted outside. Data collection took place between February and May in a large city in the Midwestern region of the United States. Recess periods ranged from 12 min to 40 min in length (M = 21.12 min; SD = 5.83 min) and primarily included traditional lunch recess periods. Schools maintained variable schedules, with some schools sending groups of students outside all at once, while others rotated the sessions with different children and different supervisors (e.g., only first through third graders at recess one, followed by only fourth and fifth graders at recess two).

Outcome assessors arrived to the school approximately 60 min before the scheduled recess session to ensure students were properly fitted with activity monitoring devices. Each data collection period contained four study team members. Two members of the team were assigned to ensure compliance in terms of properly wearing the activity monitoring devices. The other two team members collected observational data throughout the entire recess session. Data using the GRF-OT were collected by the PI or a trained graduate student. In all cases, the recess environment was completely visible to the outcome assessor, and the outcome assessor moved throughout the playground in a discreet manner in an effort to observe patterns of interaction and behavior. Final scoring of each item was completed immediately after the recess session and took into account the aggregate patterns of behavior throughout the duration of the recess session. Data using the OPP were collected at five-minute intervals across each recess period. OPP data were then averaged to create a composite level of student engagement in different activities for each recess session.

### Data analysis

Prior to data analysis, Fitbit Flex data were screened and devices registering 0 step counts in the recess time recording were eliminated from the dataset. Time recordings of the beginning and ending of each recess session were kept to allow for specificity in data extraction when examining recess-based physical activity. Furthermore, Fitbit numbers were logged and tracked for each recess session to ensure which devices were in use for each session, and which devices were returned at the end of each recess session. Given the varying times and number of students across recess sessions, we converted physical activity data to the percent of time spent in MVPA or light physical activity (LPA) during recess and used these percentages as the dependent variable in primary analyses.

Primary analyses were conducted using Hierarchical Linear Modeling (HLM) with children nested within recess sessions. Intercepts freely varied across recess sessions, while all primary predictors were entered as fixed effects. An unconditional nested model was first tested to examine possible recess-level effects for all dependent variables (i.e., physical activity levels). Next, models were fitted in which recess quality score indicators (i.e., adult engagement and supervision, student behaviors, safety and structure) were entered as predictors of physical activity levels while controlling for school as a fixed effect. Moderation was also tested by examining the interaction between gender (a level one predictor) and recess quality indicators (a level two predictor) on levels of physical activity during recess. Significant interactions were probed to examine the simple slopes and intercepts as a function of gender using the formula depicted below and as described by Preacher and colleagues [28], where ý_00_ represents the intercept, ý_01,_ ý_10_ and ý_11_ are the regression coefficients, x represents the focal predictor, and w represents the moderator variable.

$$ {\mathrm{y}}_{\mathrm{ij}}={\gg}_{00}+{\gg}{\mathrm{y}}_{10}{\mathrm{x}}_1+{{\mathrm{y}}}_{01}{\mathrm{w}}_1+{\gg{\mathrm{y}}}_{11}{\mathrm{w}}_1{\mathrm{x}}_1 $$

Finally, trends across recess quality were examined relative to high- and low-quality recess sessions (i.e., one SD above and one SD below the sample mean). In general, high-quality recess sessions were characterized by safe physical environments (e.g., lack of hazardous materials), a broad range of equipment and activities to engage in play, prosocial student behaviors (e.g., initiating games, positive communication, lack of physical violence) and present and engaged adults. In contrast, low-quality recess sessions were often characterized as unsafe environments (e.g., glass and hazardous debris), limited or no equipment to use for game play, verbal and physical conflicts, and disengaged adults. Aggregate profiles of recess sessions in the upper and lower quartile for GRF-OT scores were created to examine differential patterns in the games and activities in which children participate in, and the psychological need satisfaction of children during recess.

## Results

Descriptive statistics were calculated for all variables under study and can be found in Table 1. Results of the null models can be found in Table 2 (MVPA) and Table 3 (LPA).

**Table 1:** Descriptive statistics

Variable	Mean	SD	Range (possible range)
Total Recess Quality Score	41.15	7.44	19–54 (14–56)
Safety and Structure of Environment	14.96	3.49	6–20 (5–20)
Adult Engagement and Supervision	10.61	2.49	5–16 (4–16)
Student Behaviors	15.58	3.37	5–20 (5–20)
Physical Activity			
Percent of time spent in MVPA	50.54%	35.96%	0–100%
Percent of time spent in LPA	36.42%	29.20%	0–100%
Psychological Need Satisfaction	15.90	2.85	4–20 (4–20)
Engagement in Recess Activities (percent of students)			
Equipment	0%	1%	0–8%
Organized activities	30%	24%	0–92%
Anti-social behavior	1%	3%	0–22%
Non-engaged in play	30%	22%	0–84%
Nature	2%	6%	0–34%
Running/chasing games	17%	15%	0–76%
Traditional playground games	16%	14%	0–68%
Rough and tumble play	2%	7%	0–52%

**Table 2:** Unconditional nested model for percent of time spent in MVPA during recess

	Estimation of covariance parameters					
Parameter	Estimate	s.e.	Wald Z	***p***-value	95% CI of the estimate	ICC
Residual	.122	.003	40.99	<.001	0.116, 0.128	
Intercept Variance (Recess Session)	.009	.002	3.97	<.001	.005, .014	0.066

**Table 3:** Unconditional nested model for percent of time spent in LPA during recess

	Estimation of covariance parameters					
Parameter	Estimate	s.e.	Wald Z	***p-***value	95% CI of the estimate	ICC
Residual	.082	.002	41.00	<.001	.078, .086	
Intercept Variance (Recess Session)	.003	.001	3.59	<.001	.002, .006	0.041

**Fig. 1:**
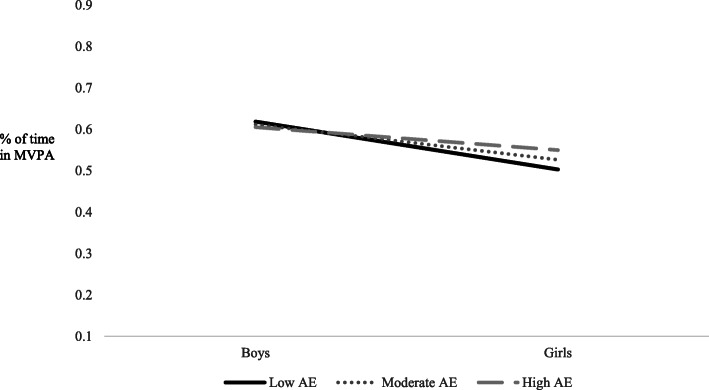
Gender x Adult engagement and supervision interaction for percentage of time in MVPA during recess

**Fig. 2:**
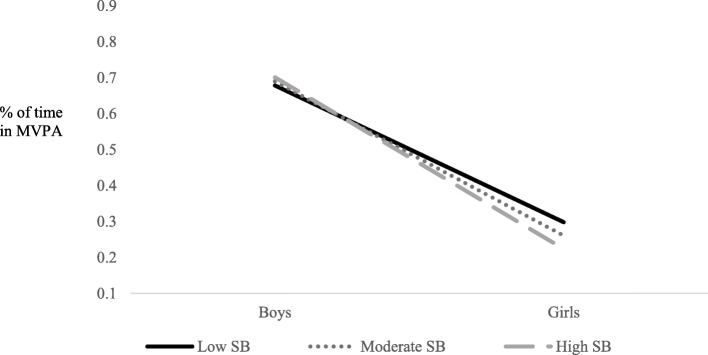
Gender x Student behavior interaction for percentage of time in MVPA during recess

An examination of predictors of the percent of time spent in MVPA at recess showed gender as the only significant predictor in the current study (*p* = .001). However, moderation analyses revealed that gender moderated the relationship between adult engagement and MVPA (*p* = .046), and student behavior and MVPA (*p* = <.001). Results can be found in Table 4. Simple slopes analyses indicated that gender was not a significant predictor of percent of time spent in MVPA at low (M-1SD; b = −.116; *p* = .107), moderate (M; b = −.085; *p* = .287) or high (M + 1SD; b = −.055, *p* = .542) levels of adult engagement and supervision. However, as can be seen in Fig. 1, the difference between boys’ and girls’ percent of time in MVPA was minimized as higher levels of adult engagement and supervision were observed. In examining student behaviors, simple slopes analyses revealed that gender was a significant predictor of percent time in MVPA at low (M-1SD; b = −.381; *p* = <.001), moderate (M; b = −.429; *p* = <.001) and high (M + 1SD; b = −.477, *p* = <.001) levels of prosocial student behavior. As can be seen in Fig. 2, boys’ percent of time in MVPA was higher at recess sessions in which more prosocial student behaviors were observed; whereas girls’ percent of time in MVPA was lower at recess sessions in which more prosocial student behaviors were observed.

**Table 4:** Estimates of effects on percent of MVPA during recess

**Estimation of covariance parameters**					
**Parameter**	**Estimate**	**s.e.**	**Wald Z**	***p-*value**	**95% CI of the estimate**
Residual	.110	.003	40.99	<.001	.105, .115
Intercept Variance (recess)	.009	.002	4.12	<.001	.006, .014
**Estimation of fixed effects**					
**Parameter**	**Estimate**	**s.e.**	***T*test statistic**	***p-*value**	**95% CI of the estimate**
Intercept	.641	.093	6.87	<.001	.454, .827
Gender	−.215	.065	−3.32	.001	−.341, −.088
School	−.002	.005	−.365	.717	−.012, .008
Safety and Structure of Environment	−.002	.006	−.380	.705	−.014, .010
Adult Engagement and Supervision	−.003	.008	−.354	.724	−.018, .013
Student behaviors	.003	.006	.519	.605	−.009, .015
Gender * Safety and Structure of Environment	.006	.004	1.32	.186	−.003, .014
Gender * Adult Engagement and Supervision	.012	.006	1.99	.046	.001, .024
Gender * Student behaviors	−.014	.004	−3.725	<.001	−.021, −.007

**Fig. 3:**
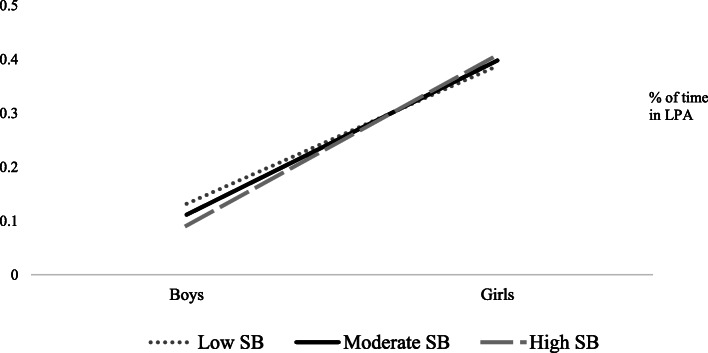
Gender x Student behavior interaction for percentage of time in light physical activity during recess

In examining LPA, gender was once again the only significant predictor of LPA (*p* = .004). Gender also moderated the relationship between student behaviors and LPA (*p* = .005). Results can be found in Table 5. Simple slopes analyses revealed that gender was a significant predictor of percent time in LPA at low (M-1SD; b = .256; *p* = <.001), moderate (M; b = .286; *p* = <.001) and high (M + 1SD; b = .317, *p* = <.001) levels of prosocial student behavior. Specifically, girls recorded higher levels of LPA during recess sessions with high levels of prosocial student behavior; whereas boys recorded lower levels of LPA during recess sessions with high levels of prosocial student behavior (see Fig. 3).

**Table 5:** Estimates of effects on percent of LPA during recess

Estimation of covariance parameters					
Parameter	Estimate	s.e.	Wald Z	***p-***value	95% CI of the estimate
Residual	.074	.002	41.00	<.001	.071, .078
Intercept Variance (recess)	.003	.001	3.60	<.001	.002, .006
**Estimation of fixed effects**					
**Parameter**	**Estimate**	**s.e.**	***T*test statistic**	***p-*value**	**95% CI of the estimate**
Intercept	.201	.063	3.21	.002	.076, .326
Gender	.152	.053	2.86	.004	.048, .257
School	.004	.003	1.18	.241	−.003, .010
Safety and Structure of Environment	.004	.005	.885	.379	−.004, .012
Adult Engagement and Supervision	.008	.005	1.52	.133	−.003, .019
Student behaviors	−.006	.004	−1.41	.163	−.014, .002
Gender * Safety and Structure of Environment	−.002	004	−.679	.497	−.009, .005
Gender * Adult Engagement and Supervision	−.008	.005	−1.50	.133	−.017, .002
Gender * Student behaviors	.009	.003	2.83	.005	.003, .015

### Engagement in recess activities

**Fig. 4:**
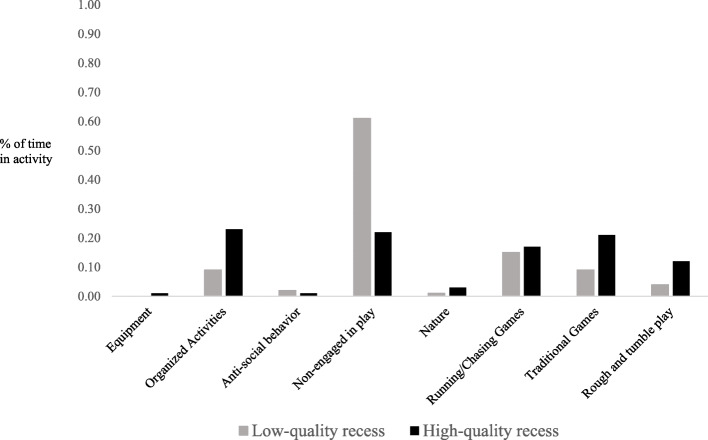
Differences in girls’ engagement in play at high- versus low-quality recess sessions

**Fig. 5:**
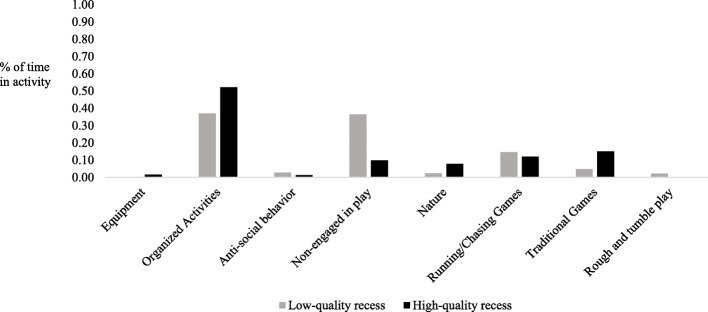
Differences in boys’ engagement in play at high- versus low-quality recess sessions

Following analyses of physical activity at recess, patterns of play were compared for recess sessions at least one standard deviation above the mean recess quality score and at least one standard deviation below the mean recess quality score (*n* = 952 boys; *n* = 952 girls). As can be seen in Fig. 4, the largest differences were seen in non-engagement in play (e.g., talking with friends, watching others), with 61% of girls at low-quality recess sessions non-engaged in play as compared to 22% of girls at high quality recess sessions. Girls at high quality recess sessions also participated in more organized games than girls at low-quality recess sessions (e.g., dance, kickball, soccer; 23% vs. 9%), and more traditional playground activities (e.g., four-square, jump ropes; 21% vs. 9%). As seen in Fig. 5, a similar pattern was observed for boys as it related to non-engagement (10% at high quality recess sessions vs. 36% at low quality recesses), participation in organized activities (52% at high quality recess sessions vs. 37% at low quality recesses), and participation in traditional playground activities (15% at high quality recess sessions vs. 5% at low quality recesses).

Finally, basic psychological need satisfaction scores were compared for those attending high-quality recess sessions (i.e., at least one SD above the mean) and those attending low-quality recess sessions (i.e., at least one SD below the mean). Results indicate that children at a high-quality recess session report higher levels of basic psychological need satisfaction at recess than children at a low-quality recess session (*t*(564) = 3.226; *p* = .001).

## Discussion

The primary purpose of the current study was to examine how contextual features of the playground impacted physical activity levels, and how this was moderated by gender. A secondary purpose was to examine patterns of play at recess to better understand how the quality of the environment shapes engagement in specific activities during this discretionary time period. Results of the current study showed that the quality of the recess environment, and the interactions of both adults and students in that environment, need to be taken into consideration in future school-based recess studies. In examining patterns of MVPA, results showed that higher levels of adult engagement and supervision reduced the disparity between boys’ and girls’ physical activity levels at recess. This finding is supported by previous research that shows higher levels of adult engagement predict higher levels of student engagement at recess [21], that children report teacher engagement as a positive influence on recess [29], and that teacher support is a facilitator of activity at recess [30]. Moreover, given previous reports of social barriers girls face in being physically active at recess (e.g., boys dominating equipment and space; 15,16), it is likely that engaged adults on the playground help to facilitate equity in access to playground resources during recess.

An examination of physical activity levels at various levels of prosocial student behaviors showed that boys participated in higher levels of MVPA during recess sessions with high pro-social behaviors. For boys in a high-quality recess session, higher levels of organized games (i.e., soccer, basketball, football) might have accounted for higher levels of MVPA. Previous research has shown higher levels of MVPA when boys participate in supervised, organized, and equipped activities [13]. In the current study, when prosocial behaviors were rated as higher (i.e., less fighting, limited arguments, high levels of game initiation, high levels of conflict resolution) games were less uninterrupted, which may have allowed boys to engage in higher levels of MVPA during recess. In contrast, girls participated in higher levels of MVPA during recess sessions with low pro-social behaviors. This finding was contrary to expectations. It is plausible that low-quality recess sessions are more chaotic, and thus might lead to more intermittent bouts of MVPA. For example, Massey and colleagues [10] reported high levels of physical altercations in urban elementary schools, which might account for some levels of MVPA. However, more research is needed to better understand this relationship. In considering overall activity patterns, popular activities for girls at high quality recess sessions included traditional playground games (e.g., 4-square, hopscotch) as well as organized activities (e.g., dance). Previous data have suggested girls view recess as a time for socialization, whereas boys primarily view recess as a time for competition and physical activity [31]. These previous findings might explain the differences observed in the intensity level of physical activity during recess. As shown in Fig. 3, LPA was higher for girls at recess sessions with high levels of pro-social behavior. These data are consistent with previous data of boys’ and girls’ play patterns at recess [15, 16, 21, 22], yet also underscore the importance of examining the context in which physical activity occurs. Notably, while increased levels of PA remains a goal of many researchers, data from the current study, as well as data reported by others [32, 33] underscore the need to understand the quality of the PA environment as it relates to children’s holistic development.

Previous research has also examined psychological need satisfaction and motivation for physical activity at recess. Notably, Stellino and Sinclair [27] reported that psychological need satisfaction was predictive of physical activity motivation at recess, and that autonomy was predictive of physical activity during recess. Results of the current study support these data, and further suggest that a high-quality recess environment can help facilitate basic psychological need satisfaction. These findings suggest that organizing the recess environment to include a variety of play opportunities and training adults to be actively engaged in recess can help satisfy student’s basic psychological needs during discretionary breaks in the school day.

### Strengths and limitations

Taken together, the major strengths of this study include a multifaceted examination of recess within an under-researched population – urban elementary school students in low income schools. Further, the results of this study shed important light on future recess research. Notably, the equivocal findings of various interventions on physical activity promotion and social behaviors [34–36] warrant a more nuanced understanding of recess, particularly in under-resourced communities. The current study suggests the need for multi-faceted interventions that concurrently focus on increased access to recess, increased access to equipment and play spaces, positive and encouraging adult behavior, and pro-social student behavior. Indeed, perhaps short-comings in previous intervention studies include a reliance on singular or dual purpose interventions, rather than a focus on multiple interventions taking place simultaneously.

The major limitations in the current study are the reduced sample size due to analysis taking place at the group level, the cross-sectional nature of the data collection, a lack of inter-rater reliability data specific to the current study, and limitations with the use of Fitbits to capture objective physical activity in child populations. Because children engage in more intermittent bouts of PA, particularly during recess, shorter measurement intervals are thought to provide more accurate estimates of time spent in various PA intensities. In a study examining differences in MVPA for children during physical education classes, McClain and colleagues [37] compared direct observation and accelerometry at various epoch lengths. Results showed that estimates of MVPA were lower when using accelerometry, as compared to direct observation regardless of epoch length. However, longer epoch lengths were associated with lower MVPA counts. Similarly, more recent research has shown lower estimates of MVPA in children when using a 60 s epoch as opposed to a 15- or 5- s measurement period [38]. Moreover, Banda and colleagues [35] reported that increased epoch length may over-estimate LPA in children as increased epoch time was associate with sedentary behavior being reclassified as LPA. Given this, it is possible that MVPA was under-estimated based on the duration and intensity of various activities engaged in during recess; whereas LPA may have been over-estimated. However, concurrent observational data of engagement in recess activities supports the overall pattern of results; specifically, higher levels of engagement in activities associated with increased PA during high- as opposed to low-quality recess sessions.

## Conclusions

Recess remains a critical opportunity for children to be physical active during the school day. Results of the current study suggest that increasing adult engagement and facilitating higher levels of pro-social behavior are important to not only physical activity promotion at recess, but also children’s psychological need satisfaction.

## Data Availability

The datasets used and/or analysed during the current study are available from the corresponding author on reasonable request.

## Abbreviations

PA: Physical Activity
US: United States
PE: Physical Education
MVPA: Moderate-to-Vigorous Physical Activity
GRF-OT: Great Recess Framework – Observational Tool
LPA: Light Physical Activity
OPP: Observations of Playground Play
BPNS: Basic Psychological Need Satisfaction
HLM: Hierarchical Linear Modeling
SD: Standard Deviation
